# Exposure of Eurasian blackbird (*Turdus merula*) to *Toxoplasma gondii* in an urban area in Thuringia, Germany

**DOI:** 10.1016/j.ijppaw.2025.101060

**Published:** 2025-03-24

**Authors:** Mike Heddergott, Rainer Hunold, Natalia Osten-Sacken

**Affiliations:** a Department of Zoology, Musée National d'Historire Naturelle, 2160 Luxembourg, Luxembourg; b Working Group Ornithology Eichsfeld, 37308, Heilbad Heiligenstadt, Germany; c Institute for Veterinary Sciences, Nicolaus Copernicus University, ul. Gagarina 7, 87-100, Toruń, Poland

**Keywords:** Eurasian blackbild, *Turdus merula*, *Toxoplasma gondii*, Urban, Wildlife, Zoonotic

## Abstract

*Toxoplasma gondii* is a zoonotic, globally distributed, obligate intracellular protozoan. Within the context of the ‘One Health’ approach, studies on toxoplasmosis are essential as it affects humans as well as domestic and wild animals, including birds. The Eurasian blackbird (*Turdus merula*) is one of the most common songbird species in Germany and previous studies have shown that they can be infected with *T. gondii*. The aim of the present study was to analyze *T. gondii* exposure in an urban blackbird population in Germany. Between 2018 and 2022, we collected fresh blackbird carcasses from an urban population in Heilbad Heiligenstadt, a small town in the German state of Thuringia. Altogether 112 blackbirds were analyzed for the presence of antibodies using a commercial indirect modified agglutination test (MAT) and parasite DNA (qPCR; brain and heart). The present study reports a high *T. gondii* seroprevalence as antibodies were detected in 50.9 % (57/112; 95 % CI: 41.6–60.2 %) of the urban blackbirds. However, *T. gondii* DNA was not detected in any of the samples analyzed. Body weight was identified as a risk factor, with heavier birds, both juveniles and adults, being more likely to test positive. Additionally, there was a significant interaction between body weight and sex. Our results suggest that *T. gondii* infection is widespread in the urban blackbird population, indicating a high parasite circulation in the environment.

## Highlights

• This is the first study on the exposure of the Eurasian blackbird (*Turdus merula*) *Toxoplasma gondii* in Germany.
• The investigations were carried out using genetic and serological methods.
• Genetically, *T. gondii* could not be detected and the seroprevalence was >50 %.
• The study indicates a high circulation of this protozoan in urban ecosystems.

## Graphical abstract

**Figure:**
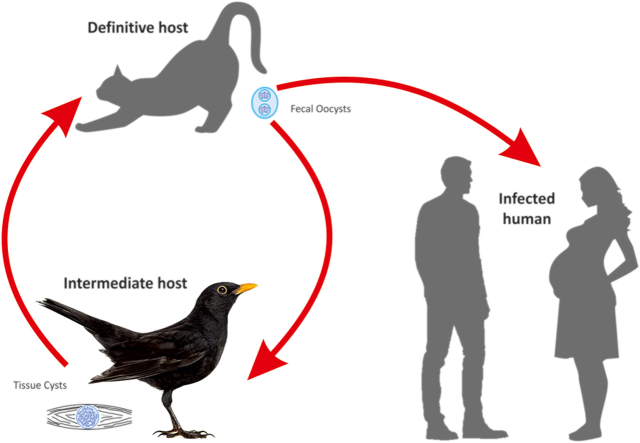

## 1 Introduction

*Toxoplasma gondii* is considered to be one of the most successful and important parasites worldwide (Dubey, 2021). The single-celled parasite can infect all warm-blooded animals including birds and is the causative agent of human toxoplasmosis, one of the most common parasitic zoonoses in the world (Tenter et al., 2000). It is assumed that one third of the world's population is chronically infected (Saadatnia and Golkar, 2012). A study conducted in Germany found antibodies to *T. gondii* in 55 % of the population (Wilking et al., 2016). *T. gondii* has an indirect life cycle in which Felidae are the definitive host and excrete the oocysts into the environment via faeces. The main route of infection is via the faecal-oral route. Infection occurs through the ingestion of water or food contaminated with sporulated *T. gondii* oocysts or through the consumption of tissue from animals infected with tissue cysts (bradyzoites) (Dubey, 2021).

Birds can become infected with *T. gondii* either by ingesting sporulated oocysts present in the environment, cysts in the tissues of infected prey, or by drinking water contaminated with oocysts. Carnivorous birds, in particular, are important indicators of the spread of *T. gondii* in prey species (Dubey et al., 2010; Molina-López et al., 2012), with scavengers and birds of prey believed to have a higher likelihood of exposure to *T. gondii* infection compared to herbivores (Dubey, 2008). It is known that *T. gondii* infection can cause disease in wild bird species, depending on the susceptibility of the host, its physical condition, and the genotype of the parasite (Dubey, 2002; Wendte et al., 2011). Clinical manifestations of infection in birds include neurological, ocular, and pulmonary diseases, or involvement of multiple organs (Wendte et al., 2011).

The Eurasian blackbird (*Turdus merula*) is a common breeding bird found throughout Europe, North Africa, Asia, and Australia (Kentish et al., 1995; Lu, 2005; Wysocki, 2006; Segelbacher et al., 2008; El Hassani et al., 2021). It is one of the most widespread passerine birds in the western Palearctic (Siriwardena et al., 1998). Blackbirds inhabit a wide range of breeding environments, from forested areas and farmland to urban settings (Zeraoula et al., 2016; Taberner et al., 2012; Figuerola et al., 2024), making them one of the most abundant bird species in cities and urban parks across the Western Palearctic today. They have a generalist diet that includes both animal and plant matter (Snow, 1988; Iglesias et al., 1993).

In the past, studies on *T. gondii* infections in Germany have primarily focused on domesticated birds (e.g., Maksimov et al., 2011; Schares et al., 2017), with only one study examining wild birds (Niederehe, 1964). Since *T. gondii* infections in blackbirds have been reported previously (Dubey, 2002), the aim of the present study was to determine the prevalence and risk factors associated with *T. gondii* infection in an urban blackbird population in Heilbad Heiligenstadt, Thuringia, Germany.

## 2 Material and methods

### 2.1 Ethics statement

All birds were found dead and made available to the authors for this study. There was no longer any special protection status and an extraordinary licence was no longer required.

### 2.2 Study area

The small town of Heilbad Heiligenstadt (51°20′ N/10°8′ E) is situated in a rural region in the northwestern part of the federal state of Thuringia, central Germany. The urban area, characterized by dense development and adjoining allotment gardens, covers an area of 7 km^2^. Due to its status as a spa town, there are also numerous green spaces and parks. The town has an average elevation of 255 m above sea level, with an annual rainfall of 839 mm. The average monthly temperature ranges from 0.4 °C in winter to 17.9 °C in summer.

### 2.3 Sample collection

**Fig. 1:**
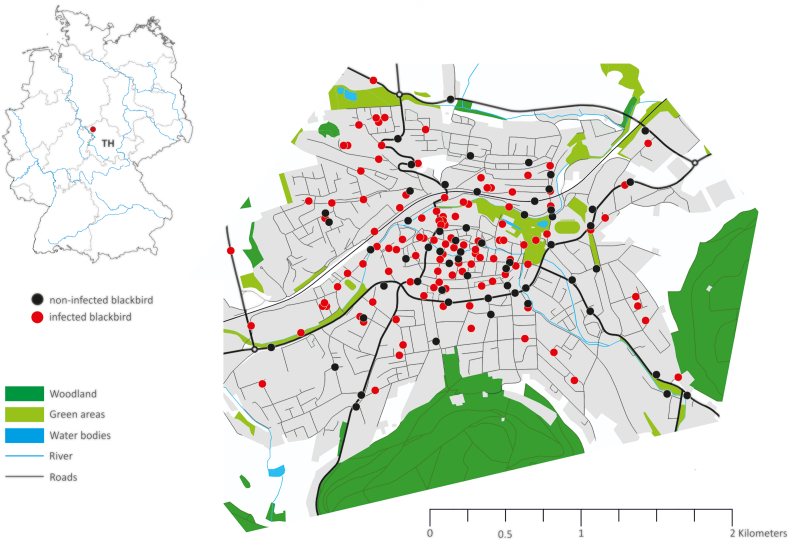
Geographical distribution of Eurasian blackbirds (*Turdus merula*) analyzed in this study and tested positive or negative for *Toxoplasma gondii* from the city of Heilbad Heiligenstadt in Thuringia (TH), Germany.

Between January 2018 and December 2022, residents of the city collected recently deceased passerine birds (Passeriformes), including 112 blackbirds, with details of their exact geographical origin (Fig. 1). Of these blackbirds, 53 (47.3 %) were killed by cats, 36 (32.1 %) were struck by vehicles, 18 (16.1 %) collided with windows, two (1.8 %) were accidently trapped indoors, one (0.9 %) drowned in a rain barrel, and for two birds (1.8 %), the cause of death was unknown. The weight of all birds was recorded using a spring scale (Bosche GmbH & Co. KG, Damme, Germany) with an accuracy of ±0.5 g. During necropsy, blood samples were taken from the heart or thoracic cavity and centrifuged at 1000×*g* for 15 min using an EBM 200 benchtop centrifuge (Hettich GmbH & Co. KG, Tuttlingen, Germany). The serum obtained was stored at −20 °C until final analysis (Heddergott et al., 2017; Osten-Sacken et al., 2024). For the genetic analysis, the brains of 81 blackbirds and the hearts of 54 blackbirds (from 23 birds brain and heart) were removed and also stored at −20 °C until analysis. Only fresh and intact tissue samples were used. Age and sex were determined based on plumage characteristics (Winkler and Jenni, 2007) and through dissection. The dataset analyzed in this study consisted of 59 males and 53 females, and 62 adults and 50 juveniles.

### 2.4 Laboratory analysis

Sera were tested for antibodies against *T. gondii* using an indirect modified agglutination test (MAT) with a commercial kit (Toxo-Screen DA®, bioMérieux, Lyon, France), including negative and positive control samples, according to the manufacturer's instructions. Among available serological tests, the MAT is widely used for diagnosing toxoplasmosis in domestic and wild birds (Dubey et al., 2021). We tested at dilutions of 1:25, 1:50, 1:100, and 1:500, with a cut-off titer of 1:25 chosen to maximize the sensitivity and specificity of the test (Dubey et al., 2010). Sera with a titer of 1:25 or higher were considered positive, and those with inconclusive results were retested.

DNA was extracted from heart and brain tissue samples (50 mg each) using a commercial kit, NucleoSpin Tissue (Macherey-Nagel, Düren, Germany), according to the manufacturer's instructions. Extracted DNA was analyzed by real-time PCR (rtPCR) using Toxo-SE (5′ AGGCGAGGGGTGAGGATGA 3′) and Toxo-AS (5′ TCGTCTCGTCTGGATCGCAT 3′) primers and a probe (5′ 6FAM-CGACGAGAGTCGGAGAGGGAGAAGATGT-BHQ1 3′), with a commercial kit (TaqMan PCR Master Mix; Applied Biosystems, Carlsbad, CA, USA). The primers Toxo-SE and Toxo-AS target the 539 bp region of *T. gondii* (REP529, GenBank no. AF146527). Positive DNA control samples were included in each run, with DNA extracted from *T. gondii* oocysts (Facultad de Veterinaria, Universidad Complutense de Madrid, Spain). The qPCR method employed is highly sensitive, capable of detecting *T. gondii* DNA from a single cyst (Galal et al., 2019).

### 2.5 Statistical analysis

We estimated the 95 % confidence interval (CI) for the prevalence estimates using the software SPSS v.22 (SPSS Inc., Chicago, IL, USA). We tested for the effect of weight, sex and age on the presence of *T. gondii* antibodies by fitting a logistic regression with linear mixed-effects models (GLMMs) in the glmmTMB package (Brooks et al., 2017). We tested for multicollinearity by estimating the variation inflation factors (VIFs) with the full GLMMs without interactions and random effects. We considered the independent variables to exhibited no significant correlation when VIF values were <5 (James et al., 2013). Given the relatively small size of the dataset as well as to avoid convergence and fitting problems, we limited the analysis to two-way interactions. We employed the dredge() function in the MuMInv.1.46.0 R package (Bartoń, 2022) to estimate Akaike information criterion (AICc) values for all potential models. When then only considered those models whose AICc values were within 2 of the model with the lowest AICc. The Statistical analyses were performed in program R v.4.2.2 (R Core Team, 2023). The marginal effects of the most parsimonious models were plotted using the plot_model() function in the sjPlot v.2.8.10 R package (Lüdecke, 2021).

## 3 Results

We found antibodies to *T. gondii* in half (50.9 %) of the examined blackbirds (57/112; 95 % CI: 41.6–60.2 %) from the urban area of Heilbad Heiligenstadt (Table 1; Fig. 1). Positive results were found at titres between 1:25 (45.7 %), 1:50 (29.8 %), 1:100 (17.4 %) and 1:500 (7.1 %). *T. gondii* DNA was not detected in any of the 81 brains or 54 hearts that were analyzed.

**Table 1:** Seroprevalence of *Toxoplasma gondii* in Eurasian blackbird (*Turdus merula*) by sex, age and collection year from the City of Heilbad Heiligenstadt in Thuringia, Germany.

Variable	Category	No. tested	No. positiv	Prevalence in % (95 % CI)^a^
Sex	Male	59	30	50.8 (37.34–63.06)
	Female	53	27	50.9 (37.30–64.50)
Age	juvenil	50	17	34.0 (20.72–47.28)
	Adult	62	40	64.5 (52.50–76.50)
Collection year	2018	18	10	55.6 (31.99–79.21)
	2019	22	10	45.5 (24.09–66.71)
	2020	21	10	47.6 (25.70–69.49)
	2021	27	14	51.9 (32.66–71.14)
	2022	24	13	54.2 (33.84–74.56)
Total		112	57	50.9 (41.60–60.20)

a CI confidence intervals.

**Fig. 2:**
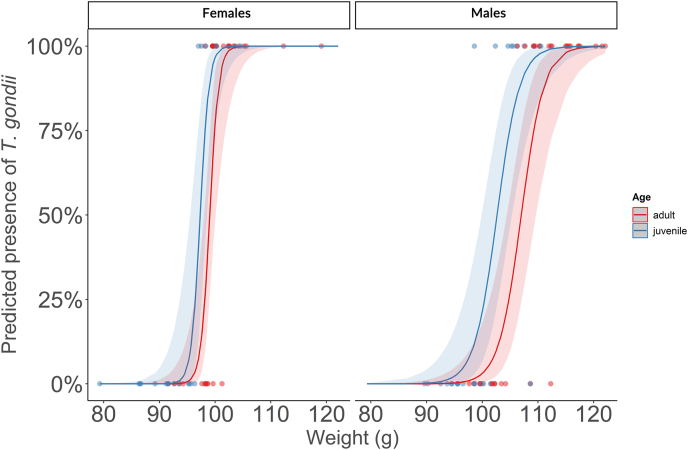
Marginal effects plots of a logistic regression model predicting the presence of *Toxoplasma gondii* antibodies in Eurasian blackbirds (*Turdus merula*) from the city of Heilbad Heiligenstadt in Thuringia, Germany, as a function of weight, sex and age category of the host. The 95 % confidence intervals are shown and the plot is based on the best-supported model identified after model selection (see Table 2).

Our model selection procedure resulted in a single best model based on the AICc. According to this model, heavier hosts were more likely to be seropositive, as were juveniles compared to adults (Table 2; Fig. 2). There was also a significant interaction between weight and sex (Table 2). Males needed to be heavier than females before they likely became seropositive (Fig. 2). Our model had a relatively high explanatory power (m*R*^2^ = 0.911).

**Table 2:** Logistic regressions identifying predictors for the presence of *Toxoplasma gondii* antibodies in Eurasian blackbirds (*Turdus merula*) from the city of Heilbad Heiligenstadt in Thuringia, Germany. Results are presented for the best-supported model identified after model selection. In the initial model, we included sex, age (juvenile vs. adult) and weight as fixed factors. We only included two-way interactions.

Coefficients	Estimate	s.e.	*z*-value	*p*-value
(Intercept)	−123.67	38.57	−3.206	0.001
Age–juvenile	2.14	0.90	2.372	0.018
Sex–males	71.12	37.00	1.922	0.055
Weight	1.25	0.39	3.205	0.001
Sex–males∗Weight	−0.76	0.38	−2.019	0.044

In the case of the continuous predictor variable (weight), the logistic regression coefficient gives the change in the log odds of seroprevalence for a one-unit increase in weight. In the case of the categorical variables, the logistic regression coefficient gives the change in the log odds of seroprevalence when considering males and juveniles relative to females and adults, and collection year respectively.

## 4 Discussion

Human activities create ideal conditions for certain domestic animals, leading to a regional overabundance of specific species, such as domestic cats, and their associated pathogens, including *T. gondii* (Medina et al., 2011). Understanding the dynamics of *T. gondii*, particularly in urban environments, requires knowledge of various factors that may influence its local distribution (Escudero et al., 2024). This study is the first to provide data on *T. gondii* prevalence in blackbirds from a small town in central Germany.

This is the second study to investigate the prevalence of *T. gondii* infections in wild birds in Germany (Niederehe, 1964) and the first study on blackbirds in Germany (Dubey, 2002; Dubey et al., 2021). The only previous study on wild birds in Germany used the dye test (DT) and reported a seroprevalence of 7.6 % in 14 tested barn owls (*Tyto alba*) and of 2 % in 49 tested rock pigeons [(*Columba livia*) = feral pigeons (*Columba livia domestica*)] (Niederehe, 1964). To date, only two studies have reported data on *T. gondii* infections in blackbirds, where the parasite was isolated from tissues of naturally infected wild individuals. In these studies, *T. gondii* was isolated from the tissues of 1.9 % of 54 European blackbirds in the Czech Republic (Literák et al., 1992) and 25 % of 4 European blackbirds in Slovakia (Čatár, 1974).

Studies on the genetic detection of *T. gondii* in blackbirds are still lacking worldwide (Dubey, 2002; Dubey et al., 2021). We were unable to detect any *T. gondii* DNA in the brain and heart tissue of blackbirds. These results likely are indicative of a low prevalence of infection (as opposed to seropositivity) in the analyzed birds. However, prevalence of infection could be artificially low as DNA detection in tissue has a lower sensitivity than serology. Furthermore, lack of PCR-based detection may be due to random distribution of *T. gondii* tissue cysts and the small volume of samples analyzed. In addition, there is usually a low parasite load in the tissue of chronically infected animals (Hill et al., 2006).

Serological studies with comparable sample sizes (≥100 birds), also based on the MAT test and conducted on wild passerines (excluding scavengers), have reported lower prevalence rates ranging from 6.2 % to 34.3 % in magpies (*Pica pica*), house sparrows (*Passer domesticus*), and Java sparrows (*Lonchura oryzivora*) across Europe and Asia (Cong et al., 2013; Huang et al., 2019; Mancianti et al., 2020). A study conducted in Brazil on domestic sparrows, using the indirect haemagglutination assay (IHA), reported a higher seroprevalence of 60.3 % (Vilela et al., 2011). However, it should be noted that the MAT test is considered the most sensitive and specific method for detecting toxoplasmosis in birds. Historically, this test has been the most commonly used for determining seroprevalence in wild birds (Dubey et al., 2021).

The high seroprevalence we observed in blackbirds is particularly noteworthy, especially when compared to earlier studies in the surrounding area of Heilbad Heiligenstadt, which also used the MAT test but found lower values in wild ruminants (Heddergott et al. 2018a, 2018b) and an carnivores (Heddergott et al., 2017). According to these studies, 22.46 % of mouflons (*Ovis orientalis musimon*) and 29.15 % of roe deer (*Capreolus capreolus*) tested positive for *T. gondii* antibodies. In contrast, the seroprevalence in the raccoon (*Procyon lotor*), an omnivorous species, was 38.3 %. We hypothesize that this difference could be attributed to a higher environmental load of *T. gondii* oocysts in urban areas compared to the surrounding countryside. This hypothesis is supported by an unpublished 2000 survey on cats in the urban area and surrounding countryside. The survey indicated a high cat density in the city, ranging from 18 to 25 cats per square kilometer (Tierheim Heiligenstadt, 2000). According to the local animal shelter, which initiated the survey, the actual cat density could be even higher due to the significant number of stray animals. Although it is assumed that the wildcat (*Felis s. sylvestris*), which is widespread in the region, also contributes to environmental contamination with *T. gondii* oocysts as a definitive host—alongside domestic cats (Heddergott et al. 2017, 2018a, 2018b)—the overall density of domestic cats in the surrounding countryside is significantly lower, estimated at 5 to 8.5 cats per km^2^ (Tierheim Heiligenstadt, 2000). Several studies have demonstrated a positive correlation between high *T. gondii* seroprevalence in wildlife and increased cat density (Miller et al., 2002; Sepúlveda et al., 2011; Barros et al., 2018; Ribas et al., 2018; VanWormer et al., 2013).

The blackbird is an opportunistic feeder with a generalized diet that includes both animal and plant foods (Cramp, 1988; Snow, 1988), and it forages primarily on the ground. Although regional studies on the feeding ecology of blackbirds are lacking, our observations align with previous findings that earthworms (Lumbricidae) constitute a significant part of the diet, particularly during the nestling rearing period from March to July (Snow, 1955; Cramp, 1988; Török and Ludvig, 1988; Iglesias et al., 1993). Our observations also confirm that plant foods, such as fruits and berries, are the second most important dietary component (Belabed et al., 2021). Other insects (Eble, 1963) and gastropods (Török, 1981) are consumed less frequently. All these food sources are known to be either infected with *T. gondii* or capable of acting as potential mechanical spreaders of *T. gondii* oocysts (Dubey, 2021; Marques et al., 2020). Thus, it is likely that blackbirds become infected with *T. gondii* oocysts through their foraging behavior on contaminated food sources.

Our results showed that body weight had a strong influence on serostatus in the blackbirds. Heavier birds were more likely to be seropositive than birds with lower body weight. Weight was determined to be a risk factor in a large number of studies in wild animals (Osten-Sacken et al., 2024) including humans (Wilking et al., 2016). Age has also been identified as an important risk factor in a large number of animals (e.g. Dubey et al., 2021; Heddergott et al., 2024), including birds (Dubey, 2008; Iemmi et al., 2020). However, in most cases only two age classes have been considered, juveniles and adults, and body weight may be a better indicator than the age of an animal. Several studies on wild birds have found an increase in seroprevalence with age (Lopes et al., 2011; Cabezón et al., 2011; Chen et al., 2024; Naveed et al., 2019; Huang et al., 2019; Iemmi et al., 2020; Lopes et al., 2021). To our knowledge, there have been no studies to date that have tested the weight of wild birds as a risk factor. In our blackbirds, juveniles at of a specific weight were more likely to be seropositive than adults. This result is somewhat counter-intuitive, as the likelihood of contact with the parasite probably increases with age. Young birds may have a higher likelihood of encountering the parasite, as they are primarily fed earthworms - potential carriers of *T. gondii* (Bettiol et al., 2000) - during the nestling period in spring and summer in this region. According to our own observations, adult birds have a broader food spectrum during the same period, including not only animal food but also plant food such as fruit and berries. It is generally assumed that carnivorous birds have higher seroprevalence values (Wilson et al., 2020; Cabezón et al., 2011; Lopes et al., 2021). In addition, we cannot definitively rule out the possibility that the result is influenced by our relatively small sample size. Furthermore, there is no validation of MAT for the blackbird and viable *T. gondii* were isolated from wild chickens with a MAT titer of 1:5 (Dubey et al., 2016). It is therefore possible that the positive threshold used in the present study with a 1:25 dilution underestimated the likely prevalence.

We found no statistically significant differences between sexes in the blackbirds we tested, suggesting that both males and females are equally exposed to and susceptible to *T. gondii* infection. This may be due to their similar feeding habits (Naveed et al., 2019). Our results are consistent with other studies on wild birds (Lopes et al., 2011; Naveed et al., 2019). However, in a study of Java sparrows in China, females were found to be significantly more seropositive than males (Huang et al., 2019).

In contrast to a study on kestrels (*Falco tinunculus*) in Italy, which found significant differences between sample years (Iemmi et al., 2020), our results showed no significant differences between years. Our results suggest that the diet of blackbirds was comparable in the years studied. These results also suggest that the environment is equally contaminated with this parasite in the years studied.

Birds are important intermediate hosts in the life cycle of *T. gondii*, potentially acting as a key source of infection for many feline definitive hosts (Gilot-Fromont et al., 2012). Each year, an estimated one billion birds are preyed upon and consumed by cats (Loss et al., 2013). Nearly half of the blackbirds analyzed in this study were preyed upon by cats, suggesting that blackbirds may significantly contribute to the maintenance of *T. gondii* in urban areas.

To our knowledge, this study is the first to investigate the prevalence of *T. gondii* infection in blackbirds in Germany and the first serological and genetic study on this bird species worldwide. Our results showed a high seroprevalence of *T. gondii* in an urban population, indicating a significant prevalence of this parasite in the urban ecosystem. This high seroprevalence suggests that blackbirds could serve as effective sentinels to monitor the presence of *T. gondii* oocysts in urban areas. Although blackbirds themselves may not play a major role in the epidemiology of *T. gondii*, they could contribute to the maintenance of the parasite in the ecosystem, particularly as prey for cats.

## CRediT authorship contribution statement

**Mike Heddergott:** Writing – review & editing, Writing – original draft, Visualization, Validation, Resources, Project administration, Methodology, Formal analysis, Data curation. **Rainer Hunold:** Writing – review & editing, Resources, Methodology, Formal analysis. **Natalia Osten-Sacken:** Writing – review & editing, Supervision, Methodology, Conceptualization.

## Declaration of competing interest

The authors whose names are listed immediately below certify that they have NO affiliations with or involvement in any organization or entity with any financial interest (such as honoraria; educational grants; participation in speakers’ bureaus; membership, employment, consul-tancies, stock ownership, or other equity interest; and expert testimony or patent-licensing arrangements), or non-financial interest (such as personal or professional relationships, affiliations, knowledge or beliefs) in the subject matter or materials discussed in this manuscript.
